# Feasibility of 10-Minute Delayed Hepatocyte Phase Imaging Using a 30° Flip Angle in Gd-EOB-DTPA-Enhanced Liver MRI for the Detection of Hepatocellular Carcinoma in Patients with Chronic Hepatitis or Cirrhosis

**DOI:** 10.1371/journal.pone.0167701

**Published:** 2016-12-09

**Authors:** Inhwan Jeon, Eun-Suk Cho, Joo Hee Kim, Dae Jung Kim, Jeong-Sik Yu, Jae-Joon Chung

**Affiliations:** 1 Department of Radiology, Yonsei University College of Medicine, Gangnam Severance Hospital, Seoul, Korea; 2 Department of Radiology, CHA University, CHA Bundang Medical Center, Seongnam-si, Korea; Yonsei University College of Medicine, REPUBLIC OF KOREA

## Abstract

**Objectives:**

To compare 10-minute (min) delayed hepatocyte phase imaging (HPI) using a 30° flip angle (FA) (10m-FA30) and 20-min delayed HPI using a 10° FA (20m-FA10) or 30° FA (20m-FA30) in Gd-EOB-DTPA-enhanced MRI in patients with chronic hepatitis or cirrhosis, in terms of lesion-to-liver contrast-to-noise ratio (CNR) for hepatocellular carcinoma (HCC) and detection sensitivity for focal hepatic lesions (FHLs).

**Materials and Methods:**

One hundred and four patients with 168 HCCs and 55 benign FHLs who underwent Gd-EOB-DTPA-enhanced MRI with 10m-FA30, 20m-FA10, and 20m-FA30 were enrolled. Patients were divided into two groups according to the Child-Pugh classification: group A with chronic hepatitis or Child-Pugh A cirrhosis and group B with Child-Pugh B or C cirrhosis. Lesion-to-liver CNR for HCCs was compared between 10m-FA30 and 20m-FA10 or 20m-FA30 for each group. The presence of FHLs was evaluated using a four-point scale by two independent reviewers, and the detection sensitivity was analyzed.

**Results:**

In group A, the CNR for HCCs (n = 86) on 10m-FA30 (165.8 ± 99.7) was significantly higher than that on 20m-FA10 (113.4 ± 71.4) and lower than that of 20m-FA30 (210.2 ± 129.3). However, there was no significant difference in the sensitivity of FHL detection between 10m-FA30 (mean 95.0% for two reviewers) and 20m-FA10 (94.7%) or 20m-FA30 (94.7%). In group B, the CNR (54.0 ± 36.4) for HCCs (n = 57) and the sensitivity (94.2%) of FHL detection for 10m-FA30 were significantly higher than those for 20m-FA10 (41.8 ± 36.4 and 80.8%, respectively) and were not different from those for 20m-FA30 (62.7 ± 44.4 and 93.3%, respectively).

**Conclusion:**

The diagnostic performance of 10m-FA30 was similar to or higher than 20m-FA10 or 20m-FA30 in both groups A and B. This finding indicates that 10m-FA30 could replace 20-min delayed HPI regardless of patient liver function and reduce the delay time by 10 minutes.

## Introduction

Gadolinium ethoxybenzyl diethylenetriamine pentaacetic acid (Gd-EOB-DTPA) is a liver-specific contrast agent for magnetic resonance imaging (MRI) that provides both dynamic imaging and static hepatocyte-specific imaging. Recent reports show that Gd-EOB-DTPA–enhanced MRI is highly sensitive for the detection of focal hepatic lesions (FHLs) such as hepatocellular carcinomas (HCCs) and metastatic tumors [1–3]. However, sufficient enhancement of liver parenchyma on hepatocyte phase imaging (HPI) from adequate Gd-EOB-DPTA uptake by hepatocytes is necessary to detect FHLs [4]. HPI is usually obtained 20-minute (min) after intravenous injection of Gd-EOB-DTPA [1, 5–7]. Several studies have tried to reduce the delay time for HPI after contrast injection [8–11] because decreasing this delay time would increase patient throughput and reduce patient discomfort during MRI scanning [11]. Ten-min delayed HPI may be sufficient for detection and characterization of FHLs, but has a significantly lower signal ratio or lesion-to-liver contrast-to-noise ratio (CNR) than 20-min delayed HPI [8, 10].

HPI has commonly been obtained using a T1-weighted (T1w) three-dimensional (3D) gradient echo (GRE) sequence with a flip angle (FA) of 10–15° to match the FA used for dynamic sequences [12, 13]. However, several studies suggest that using a higher FA of 30–40° improves detection, conspicuity, and lesion-to-liver CNR for FHLs [14–17] because a higher FA increases the T1-weighting [14]. Recent studies have introduced the possibility of reducing the delay time for HPI without loss of CNR by applying higher FA [18, 19]. Five-min or 10-min delayed imaging with 30° FA (5m-FA30 or 10m-FA30, respectively) yields higher CNR and similar detection sensitivity for FHL compared to 20-min delayed HPI with 10° FA (20m-FA10). Interestingly, the CNR for HCCs was found to be significantly higher on 5m-FA30 than on 20m-FA10 [18], but lesion-to-liver CNR according to liver function was not evaluated. The hepatic uptake of Gd-EOB-DTPA is related to the severity of cirrhosis [20–22].

We hypothesized that 10-min delayed HPI using a high FA of 30° would have similar or better diagnostic performance for HCC compared with conventional 20-min delayed HPI, regardless of the degree of liver cirrhosis. We speculated that 10 minutes of delay for HPI would be sufficient for adequate hepatic uptake of Gd-EOB-DTPA in patients with chronic hepatitis or compensated cirrhosis, and noted that patients with decompensated cirrhosis display no more Gd-EOB-DTPA uptake after 10 minutes in delay time [20–22]. Therefore, we divided enrolled patients into compensated and decompensated liver disease groups [23], and compared the CNR for HCCs and detection sensitivity for FHLs between 10m-FA30 and 20m-FA10 for each subgroup. In addition, the values between 10m-FA30 and 20-min delayed HPI with 30° FA (20m-FA30) were compared. The final aim of our study was to determine whether the 10m-FA30 protocol could replace 20-min delayed HPI in patients with chronic hepatitis or cirrhosis, based on CNR of HCCs and detection sensitivity for FHLs.

## Materials and Methods

The Gangnam Severance Hospital institutional review board (IRB) approved this retrospective study and written informed consent was waived. Patient records were anonymized and de-identified prior to analysis.

### Patient Population and Standard Reference

From May 2012 to November 2014, 124 consecutive patients with suspected or known HCC underwent a Gd-EOB-DTPA-enhanced liver MRI. Twenty patients were excluded from the study because of an unknown final diagnosis owing to the lack of a valid reference standard (n = 15) and duplicate MRI examinations of the same patients (n = 5). Therefore, a total of 104 with 168 HCCs and 55 benign FHLs were analyzed (Table 1).

**Table 1:** Patient Clinical Information and Focal Hepatic Lesion Characteristics.

Mean age (age range)			61 (44–81)
Sex (M/F)			85/19
Diagnosis of FHLs	HCC		168
	Benign	Cyst	19
		Hemangioma	15
		DN/CN	21
Size of FHLs	Large	HCC	143
		Benign	30
	Small	HCC	25
		Benign	25
Background liver	Chronic hepatitis	Hepatitis B virus	16
		Hepatitis C virus	2
		Non-B and non-C	3
	Cirrhosis	Hepatitis B virus	59
		Hepatitis C virus	9
		Alcoholic	3
		Non-B and non-C	12
Child-Pugh classification		A	78
		B	21
		C	5

FHLs = focal hepatic lesions; HCC = hepatocellular carcinoma; DN/CN = dysplastic/cirrhotic nodule; Large = focal hepatic lesions 10 mm or greater in short axis; Small = focal hepatic lesions less than 10 mm in short axis; Non-B/C = non-B and non-C viral.

The final diagnostic criteria for all 168 HCCs (mean size ± standard deviation, 2.3 ± 1.7 cm; range, 0.6–9.0 cm) were as follows: pathologically proven (n = 22 by resection and n = 1 by biopsy), tumor staining on transarterial chemoembolization (TACE) with sustained iodized-oil accumulation on follow-up CT (n = 130), and typical findings on dynamic imaging (arterial hypervascularity and portal venous/delayed phase washout on dynamic CT and/or arterial hypervascularity and portal venous phase washout on dynamic MRI) (n = 15).

Among the 55 benign lesions, one cirrhotic nodule was diagnosed pathologically after liver transplantation. Diagnosis of the remaining 54 lesions, including 19 cysts (0.8 ± 0.4 cm; 0.4–1.8 cm), 15 hemangiomas (0.9 ± 0.5 cm; 0.4–2.3 cm), and 21 dysplastic or cirrhotic nodules (1.5 ± 0.6 cm; 0.8–2.8 cm), was established based on well-confirmed characteristic imaging criteria and non-progressive appearance in size on follow-up imaging studies, which included examinations before the present study period [24–27].

Pathologic specimens were used in 34 patients to confirm chronic hepatitis (n = 1 by biopsy and n = 14 by surgical specimen) and cirrhosis (n = 2 by biopsy and n = 17 by surgical specimen). Chronic hepatitis or cirrhosis of the other 70 patients was diagnosed by imaging findings using ultrasonography, CT, and MRI (liver surface irregularity, marginal dullness, atrophy of the right lobe and segment 4 of liver, hypertrophy of the left lobe and caudate lobe of liver, splenomegaly, and development of collateral veins), with referring blood examinations (prothrombin time, platelet counts, alanine aminotransferase, and aspartate aminotransferase) [20, 28–32]. Etiology of chronic hepatitis or cirrhosis is presented in Table 1.

Patients with chronic hepatitis or cirrhosis were classified into two groups according to the Child-Pugh classification: group A with chronic hepatitis or compensated liver cirrhosis (Child-Pugh class A) or group B with decompensated liver cirrhosis (Child-Pugh B or C) [23].

### MR Image Protocol

All studies were conducted using a 1.5 T MR scanner (Magnetom Avanto; Siemens Medical Solutions, Erlangen, Germany). For all patients, 3D axial fat suppressed T1w volumetric interpolated breath-hold examination (VIBE) images were acquired for dynamic imaging along with 10-min or 20-min delayed HPI after the administration of Gd-EOB-DPTA. A 30° FA was used for 10-min delayed HPI and both 10° and 30° FAs were used for 20-min delayed HPI. The other VIBE imaging parameters were as follows: repetition time (TR)/echo time (TE) of 5.1/2.4 msec, slice thickness of 2.8 mm, matrix of 256 x 179, number of excitations of 1, receiver bandwidth of 300 Hz/pixel, integrated parallel imaging techniques (iPAT) using acceleration factors of 2, k-space trajectory of rectangular (10° FA) and central (30° FA) ordering of the echo train following a spectral fat suppression pulse, and an acquisition time of 14–15 seconds at either 10° FA or 30° FA imaging. A dose of 0.025 mmol/kg of Gd-EOB-DTPA (Primovist, Bayer Schering Pharma, Berlin, Germany) was injected at a rate of 1 mL/s using a power injector (MedRad Spectris Solaris EP, Medrad, Indianola, PA, USA) through a 20-gauge catheter inserted into the veins of the antecubital area, followed by 20 mL of normal saline at the same injection rate. All MR images were sent to a picture archive and communication systems (PACS) workstation (Centricity RA1000, GE Healthcare, Milwaukee, USA) for image analysis.

### Quantitative Analysis

All HCCs were divided into two groups by size: small HCCs (<10 mm in the short-axis) or large HCCs (≥10 mm in the short-axis). The short-axis diameter of each tumor was measured on the 20m-FA10. Quantitative analysis was performed by a radiologist with three years of liver MRI experience. The signal intensity (SI) of the liver parenchyma and each HCC were measured on three HPIs (10m-FA30, 20m-FA10 and 20m-FA30). Background image noise was measured ventral to the liver outside of the patient’s body. All measurements were repeated three times for each image, and the mean value was used for assessment of the lesion-to-liver CNR as follows: CNR = (SI_Liver_—SI_HCC_)/SD_Noise_, where SI_Liver_ is the mean SI of the liver parenchyma, SI_HCC_ is the mean SI of HCC, and SD_Noise_ is the mean standard deviation of the background image noise.

### Qualitative Analysis

The three HPIs (10m-FA30, 20m-FA10 and 20m-FA30) were independently and randomly evaluated by two radiologists with 18 years and 9 years of experience in liver MRI. Three image review sessions were spaced 3–4 weeks apart to prevent memory recall bias. All images were assessed on PACS using an optimal window setting. The reviewers evaluated the presence of FHL using a four-point scale as follows: 1 = definitely absent, 2 = probably absent, 3 = probably present, 4 = definitely present. The reviewers marked FHLs using arrows and scales on PACS and a coordinator with three years of liver MRI experience matched them to the reference standard.

### Statistical Analysis

Statistical analyses were performed using standard statistical software (SPSS Version 21; IBM SPSS Statistics, IBM Corporation, Chicago, IL, USA). For the calculation of detection sensitivities for all FHLs, scores of 1 and 2 were considered negative and scores of 3 and 4 were regarded as positive. The McNemar test was used to compare the detection sensitivities for FHLs between 10m-FA30 and 20m-FA10 or 20m-FA30. The paired t-test was used to compare the lesion-to-liver CNR for large HCCs (≥10 mm in the short-axis) between 10m-FA30 and 20m-FA10 or 20m-FA30 and the image noise between 20m-FA10 and 20m-FA30. The patients were divided into groups A and B according to the Child-Pugh classification [23], and subgroup analysis was conducted as well. The correlations of between lesion-to-liver CNR for HCCs and Child-Pugh or Model for End-Stage Liver Disease (MELD) scores were analyzed using the Pearson correlation coefficient.

For both the McNemar test and paired t-test, the Bonferroni corrected p value was applied and a p-value of < 0.025 (= 0.05/2) was considered statistically significant. Inter-reader agreement was assessed using Cohen’s kappa coefficient, which was interpreted according to the guidelines of Landis and Koch [21].

## Results

**Fig 1:**
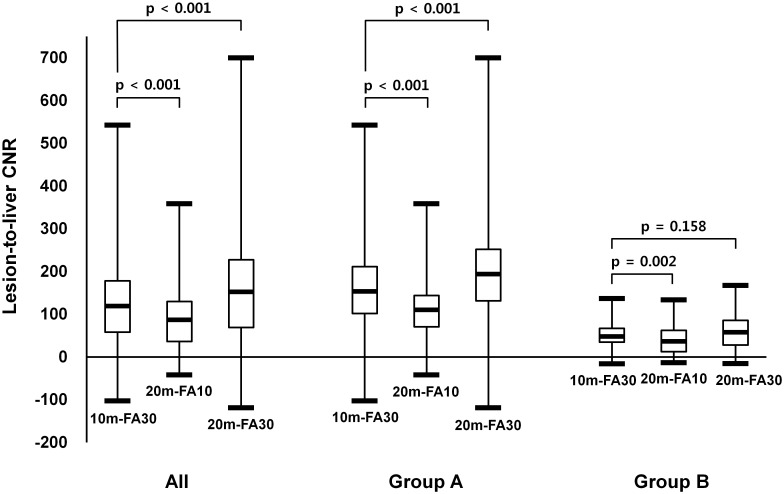
Lesion-to-liver contrast-to-noise ratio (CNR) for HCCs. Box-and-whisker plots showing the median (middle line of each box), quartiles (top and bottom lines of each box), and upper and lower adjacent (upper and lower whiskers for each box) values of the lesion-to-liver contrast-to-noise ratio (CNR) for HCCs. HCCs were imaged according to three different protocols: 10-minute delayed hepatocyte phase imaging (HPI) with a 30° flip angle (10m-FA30), 20-minute delayed HPI with a 10° flip angle (20m-FA10) or a 30° flip angle (20m-FA30). The CNR for HCCs on 10m-FA30 was significantly higher than that on 20m-FA10 and significantly lower than that on 20m-FA10, in both all patients and those just in group A (with chronic hepatitis or Child-Pugh A cirrhosis). In group B with Child-Pugh B or C cirrhosis, the CNR for HCCs on 10m-FA30 was significantly higher than that on 20m-FA10 and was not different from that on 20m-FA30.

**Fig 2:**
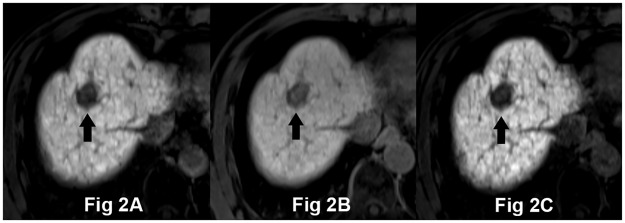
A 61-year-old man with HCC and hepatitis B-related compensated liver cirrhosis (Child-Pugh score 5). Hepatocyte phase imaging (HPI) were acquired using 30° flip angle (FA) at 10-minute delayed time (10m-FA30) (A), 10° FA at 20-minute delayed time (20m-FA10) (B), and 30° FA at 20-minute delayed time (20m-FA30) (C) after Gd-EOB-DTPA administration. These images are viewed at the same window level (400) and width (700). HCC in segment 8 appears as low signal intensity related to the surrounding liver parenchyma on all three HPIs (arrow). Lesion-to-liver CNRs on 10m-FA30, 20m-FA10, and 20m-FA30 were 222.8, 147.5, and 343.1, respectively. The two reviewers’ mean subjective scores for the presence of focal hepatic lesions were 4 (4 and 4) on all three HPIs.

**Fig 3:**
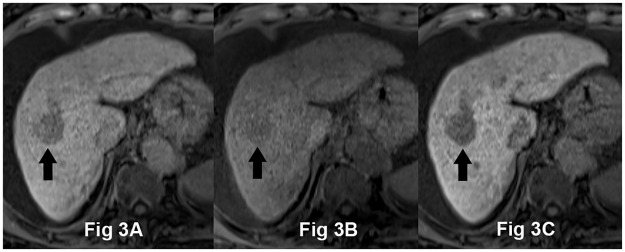
A 76-year-old woman with HCC and hepatitis C-related decompensated liver cirrhosis (Child-Pugh score 8). Hepatocyte phase imaging (HPI) were acquired using 30° flip angle (FA) at 10-minute delayed time (10m-FA30) (A), 10° FA at 20-minute delayed time (20m-FA10) (B), and 30° FA at 20-minute delayed time (20m-FA30) (C) after Gd-EOB-DTPA administration. These images are viewed at the same window level (400) and width (700). HCC in segments 5/6 appears as low signal intensity related to the surrounding liver parenchyma on all three HPIs (arrow). Lesion-to-liver CNRs on 10m-FA30, 20m-FA10, and 20m-FA30 were 35.0, 12.2, and 59.6, respectively. The two reviewers’ mean subjective scores for the presence of a focal hepatic lesion were 4 (4 and 4) on both 10m-FA30 and 20m-FA30 and 2 (2 and 2) on 20m-FA10.

The lesion-to-liver CNRs on the three HPIs are presented in Table 2 and Fig 1. The CNR for all HCCs in patients for 10m-FA30 (130.6 ± 99.6) was significantly higher than for 20m-FA10 (90.9 ± 70.8), but significantly lower than for 20m-FA30 (163.5 ± 129.7). In group A, the CNR of HCCs for 10m-FA30 (165.8 ± 99.7) was also significantly higher than for 20m-FA10 (113.4 ± 71.4) and lower than for 20m-FA30 (210.2 ± 129.3) (Fig 2). In group B, the CNR of HCCs for 10m-FA30 images (54.0 ± 36.4) was significantly higher than that for 20m-FA10 (41.8 ± 36.4), but there was no significant difference in the CNR between 10m-FA30 images and 20m-FA30 images (62.7 ± 44.4) (Fig 3). The lesion-to-liver CNR of group A was significantly higher than that of group B for each image group (p < 0.001).

**Table 2:** Mean Lesion-to-liver Contrast-to-Noise Ratio (CNR) on 10-minute Delayed Hepatocyte Phase Imaging (HPI) using a 30° Flip Angle (FA) (10m-FA30), 20-minute Delayed HPI using a 10° FA (20m-FA10), and 20-minute Delayed HPI using a 10° FA (20m-FA10).

	Lesion-to-liver contrast-to-noise ratio (CNR)				
	10m-FA30	20m-FA10	^a^*p* value	20m-FA30	^b^*p* value
All HCCs	130.6 ± 99.6	90.9 ± 70.8	< 0.001	163.5 ± 129.7	< 0.001
HCCs in group A	165.8 ± 99.7	113.4 ± 71.4	< 0.001	210.2 ± 129.3	< 0.001
HCCs in group B	54.0 ± 36.4	41.8 ± 36.4	= 0.002	61.7 ± 44.4	= 0.158

Group A = patients with chronic hepatitis or compensated liver cirrhosis (Child-Pugh A). Group B = patients with decompensated liver cirrhosis (Child-Pugh B or C).

^a^ Paired t-test between 10m-FA30 and 20m-FA10,

^b^ paired t-test between 10m-FA30 and 20m-FA30. The Bonferroni corrected p value of < 0.025 (= 0.05 / 2) is considered statistically significant.

**Fig 4:**
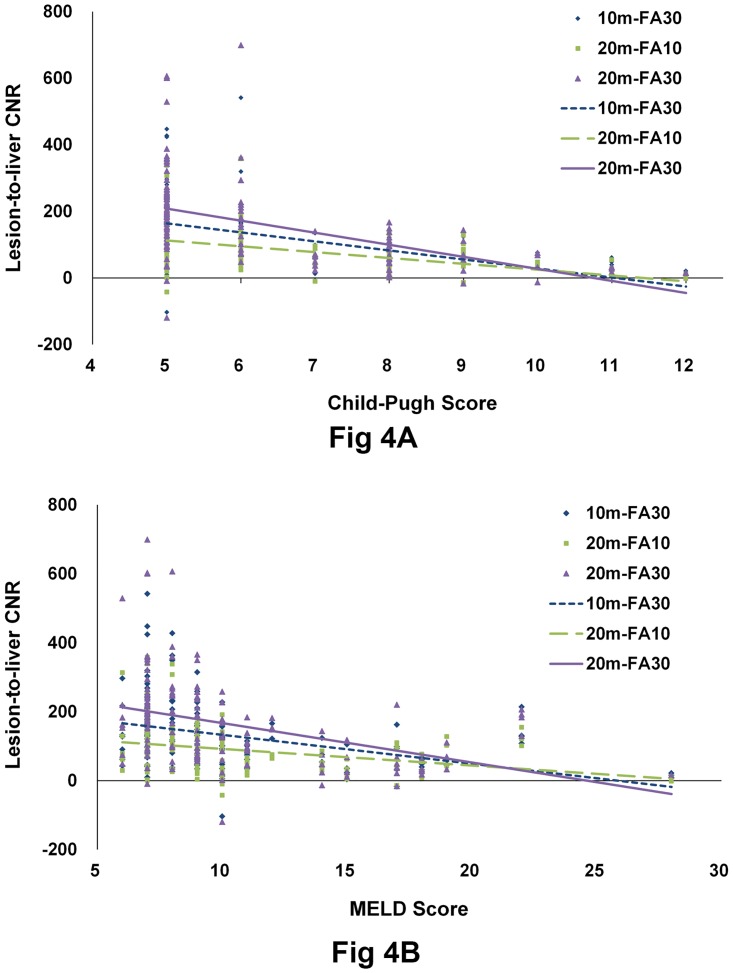
Lesion-to-liver contrast-to-noise ratio (CNR) for HCCs according to Child-Pugh and MELD scores. The relationships between CNR and Child-Pugh score (A) are as follows: y = -27.2x + 300.3 (r = - 0.491) for 10m-FA30, y = -17.5x + 200.5 (r = - 0.446) for 20m-FA10; and y = -36.3x + 390.1 (r = - 0.503) for 20m-FA30. The relationships between CNR and MELD score (B) are as follows: y = -8.4x + 217.3 (r = - 0.378) for 10m-FA30; y = -4.8x + 140.5 (r = - 0.304) for 20m-FA10; and y = -11.4x + 281.1 (r = - 0.393) for 20m-FA30.

**Fig 5:**
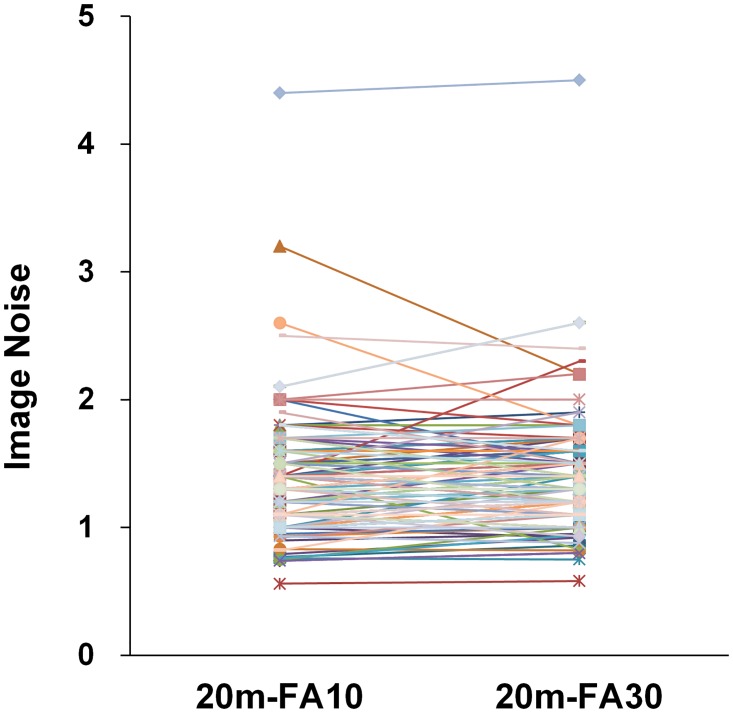
The correlation of image noise between on 20m-FA10 and 20m-FA30. There was no significant difference in noise between on 20m-FA10 (mean noise ± standard deviation, 1.33 ± 0.45) and on 20m-FA30 (1.35 ± 0.43), which suggests the image noise was not influenced when applying a 30° flip angle instead of a 10° flip angle.

There were negative relationships between the CNR of HCCs and Child-Pugh or MELD scores for the three HPIs (Fig 4). Pearson correlation coefficients between CNR and Child-Pugh score were as follows: r = - 0.491 (p < 0.001) for 10m-FA30; r = - 0.446 (p < 0.001) for 20m-FA10; and r = - 0.503 (p < 0.001) for 20m-FA30. Pearson correlation coefficients between CNR and MELD score were as follows: r = - 0.378 (p < 0.001) for 10m-FA30; r = - 0.304 (p < 0.001) for 20m-FA10; and r = - 0.393 (p < 0.001) for 20m-FA30. Paired t-tests showed there was no significant difference in noise between on 20m-FA10 and on 20m-FA30 (p value = 0.295) (Fig 5).

Detection sensitivities for FHLs on the three HPIs are summarized in Table 3. In all patients (both group A and B), the detection sensitivities for all FHLs, large FHLs (≥10 mm in the short-axis) and HCCs for 10m-FA30 were significantly higher than for 20m-FA10 for reader A. The detection sensitivity for them at 10m-FA30 for reader B tended to be higher than at 20m-FA10 although there was no statistical difference. There was no significant difference in detection sensitivity between 10m-FA30 and 20m-FA30 for both readers. In group A, there was no significant difference in the detection sensitivity between 10m-FA30 and 20m-FA10 or between 10m-FA30 and 20m-FA30 for both readers, irrespective of the lesions’ malignancy or benignity and the size of the FHLs. In group B, the detection sensitivities for all FHLs, large FHLs (≥10 mm in the short-axis) and HCCs for 10m-FA30 were significantly higher than for 20m-FA10. However, there was no significant difference in detection sensitivity between 10m-FA30 and 20m-FA30.

**Table 3:** Detection Sensitivity of Focal Hepatic Lesions for the Two Readers on 10-minute Delayed Hepatocyte Phase Imaging (HPI) using a 30° Flip Angle (10m-FA30), 20-minute Delayed HPI using a 10° Flip Angle (20m-FA10) or a 30° Flip Angle (20m-FA30).

		Reader A			Reader B		
		10m-FA30	20m-FA10	20m-FA30	10m-FA30	20m-FA10	20m-FA30
All	All lesions (223)	94.6%	^a^**91.0%**	93.7%	92.4%	89.2%	92.4%
	Large (180)	95.0%	^a^**90.6%**	95.0%	92.8%	88.9%	93.9%
	Small (43)	93.0%	93.0%	88.4%	90.7%	90.7%	86.0%
	HCC (168)	94.0%	^a^**89.3%**	93.5%	91.7%	87.5%	92.3%
	Benign (55)	96.4%	96.4%	94.5%	94.5%	94.5%	92.7%
Group A	All lesions (171)	95.9%	95.3%	95.3%	94.2%	94.2%	94.2%
	Large (131)	96.9%	96.2%	97.7%	95.4%	95.4%	96.9%
	Small (40)	92.5%	92.5%	87.5%	90.0%	90.0%	85.0%
	HCC (119)	95.8%	95.0%	95.8%	94.1%	94.1%	95.0%
	Benign (52)	96.2%	96.2%	94.2%	94.2%	94.2%	92.3%
Group B	All lesions (52)	96.2%	^a^**82.7%**	94.2%	92.3%	^a^**78.8%**	92.3%
	Large (49)	95.9%	^a^**81.6%**	93.9%	91.8%	^a^**77.6%**	91.8%
	HCC (49)	95.9%	^a^**81.6%**	93.9%	91.8%	^a^**77.6%**	91.8%

Data in parentheses = the number of focal hepatic lesions. Group A = patients with chronic hepatitis or compensated liver cirrhosis (Child-Pugh A). Group B = patients with decompensated liver cirrhosis (Child-Pugh B or C). Large = focal hepatic lesions 10 mm or greater in short axis. Small = focal hepatic lesions less than 10 mm in short axis.

^a^ Bonferroni corrected p value (Bold) is lesser than significance threshold (p value < 0.025 = 0.05/2) when the McNemar test was applied between 10m-FA30 and 20m-FA10 or 20m-FA30, but there was no significant difference in the detection sensitivity between 10m-FA30 and 20m-FA30. In group B, statistical analysis for small lesions or benign lesions could not be performed because of the small number of lesions.

The interobserver agreement was almost perfect for both readers (kappa value = 0.860).

## Discussion

In patients with compensated or decompensated liver disease, 10m-FA30 was superior to 20m-FA10 in terms of CNR for HCCs or detection sensitivity for FHLs. In patients with compensated liver disease, there was no significant difference in detection sensitivity between 10m-FA30 and 20m-FA30, even though the lesion-to-liver CNR for 20m-FA30 was higher than that for 10m-FA30. In decompensated liver disease, there was no significant difference in lesion-to-liver CNR and detection sensitivity between 10m-FA30 and 20m-FA30.

Dynamic contrast-enhanced imaging and HPI have usually been obtained using a T1w FS 3D GRE sequence with low FA and short TR to decrease scan time. Because of the relatively short TR, longitudinal magnetization is not completely recovered. This effect is more obvious for tissue with a longer T1 relaxation time such as hepatic malignant tumors without Gd-EOB-DTPA uptake, but relatively vague for normal liver parenchyma with a shorter T1 relaxation time arising from adequate Gd-EOB-DTPA uptake. The difference of residual longitudinal magnetization between hepatic malignant tumors and normal liver parenchyma is amplified by using a higher FA. Thus, increasing the FA boosts T1-weighting and improves the contrast between hepatic malignant tumors and surrounding liver parenchyma. Accordingly, higher FA has been recommended to produce a greater lesion-to-liver CNR and improve detection of FHLs [14, 16]. On the basis of this theoretical background, we attempted to use a higher FA to increase CNR for HCC and reduce the delay time for HPI while maintaining detection performance for HCC in patients with chronic hepatitis or cirrhosis.

In previous studies, lesion-to-liver contrast ratios or CNRs on HPI acquired using a higher FA (30–35°) were significantly better than those acquired using a lower FA (10–12°) at each delay time from 5 to 10 minutes [15, 16]. Another study showed that 5-min delayed transitional phase imaging using a 30° FA (5m-FA30) had a higher lesion-to-liver CNR for HCCs compared with 20m-FA10 [18]. However, most patients in that study had chronic hepatitis or compensatory liver cirrhosis (Child-Pugh A) and only 4 out of 32 patients with HCC had decompensated liver cirrhosis (Child-Pugh B or C) [18]. In addition, the CNR difference between 5min-FA30 and 20m-FA10 was not analyzed according to the severity of cirrhosis [18]. In advanced cirrhosis, lesion-to-liver CNR and liver parenchymal enhancement on HPI decrease compared with normal liver or early stage cirrhosis because the number of functioning hepatocytes and the ability of hepatocytes to uptake Gd-EOB-DTPA deteriorate [20–22, 33, 34]. Several studies have found that liver parenchymal enhancement in patients with Child-Pugh A cirrhosis gradually increases over time to 20 minutes after administration of Gd-EOB-DTPA, whereas patients with Child-Pugh B or C cirrhosis do not have a stepwise increase after the portal venous phase [20, 21]. This outcome suggests that waiting for 20 minutes to obtain HPI is not necessary in patients with advanced cirrhosis [22]. In our study, we therefore divided the patients into group A with compensated liver disease group (chronic hepatitis or Child-Pugh A cirrhosis) and group B with decompensated liver disease group (Child-Pugh B or C cirrhosis) [23] and then compared the lesion-to-liver CNR for HCC and the detection sensitivity for FHLs among the three HPIs within these subgroups.

In a previous study, patients with Child-Pugh A cirrhosis showed strong liver parenchymal enhancement on 20-min delayed HPI that was not much different than in patients without liver cirrhosis [21]. From this observation, we inferred that the impairment of hepatocyte function in early stage liver cirrhosis does not significantly change the lesion-to-liver contrast. In a previous study of patients with normal liver function, 10m-FA30 showed a higher lesion-to-liver CNR and similar detection sensitivity for metastatic tumors compared to 20m-FA10 [19]. In our study, 10m-FA30 had a higher lesion-to-liver CNR of HCCs and similar detection sensitivity compared to 20m-FA10 for group A (patients with chronic hepatitis and compensated liver cirrhosis), which was concordant with the findings of the previous study of patients with normal liver function [19]. There was also no significant difference in lesion detection sensitivity between 10m-FA30 and 20m-FA30, although the CNR of 20m-FA30 was significantly higher than that of 10m-FA30. Such results may suggest that for the detection of HCC in patients with chronic hepatitis and compensated liver cirrhosis, 20-min delayed HPIs can be replaced by 10m-FA30 with similar or better diagnostic performance, as it has a sufficiently high CNR and detection sensitivity.

In advanced liver cirrhosis, liver parenchymal enhancement deteriorated on HPI compared with normal liver or early stage cirrhosis [20–22, 33, 34]. Therefore, it has been suggested that HPI after 20 minutes or more might be required for cirrhotic patients [34, 35]. However, several studies have shown that patients with Child-Pugh B or C cirrhosis do not have an increase in liver parenchymal enhancement after 8 or 10 minutes after Gd-EOB-DTPA administration [21, 22]. The author proposed that waiting 20 minutes for HPI was not necessary, and that HPI might be obtained within 10 or 15 minutes after contrast administration to reduce MR examination time [22]. We obtained HPI after a delay of 10 minutes and applied a high FA of 30° at HPI. In group B (patients with decompensated liver cirrhosis), the CNR for HCC and detection sensitivity for FHLs on 10m-FA30 were significantly superior to those on 20m-FA10. There was no significant difference in lesion-to-liver CNR or detection sensitivity between 10m-FA30 and 20m-FA30. Therefore, we suggest that in patients with decompensated liver cirrhosis, 10m-FA30 may be better than 20m-FA10 or 20m-FA30, given the higher or similar diagnostic performance and time savings of 10 minutes.

Although using a higher FA on HPI increases the CNR of the tumor in the liver, it also enhances the energy deposition in the patient’s body by radiofrequency (RF) pulses, referred to as the specific absorption rate (SAR). The SAR, which relates to field strength, transmitter-coil type, RF power, duty cycle, and patient body size, is proportionate to the square of the strength of the FA and the static magnetic field. Accordingly, increasing the FA from 10° to 30° leads to a nine-fold increase of the SAR [14], which is usually not a problem at 1.5 T. However, it can be a considerable increase for 3.0T MRI, where the baseline SAR is four-times higher than that of 1.5 T MRI [14, 16]. In HPI using a higher FA, an increase of ghosting artifact of the bile duct was reported in the previous study [12]. Although that reason is uncertain, the ghosting artifact seems to be visually more apparent because of increased SI of the excreted contrast in the biliary duct at imaging using a higher FA [12]. We observed that subcutaneous fat was in-homogeneously suppressed at imaging with a higher FA, compared with a lower FA. With specialized ‘Quick Fat-Sat’ of the VIBE sequence used in this study, linear ordering, which also is referred as rectangular ordering, is performed as long as the fat signal along the echo train after one fat saturation pulse can be nulled in k-space center. When we increase FA, there is a point where even a 180° fat saturation pulse will not be enough. In that case the sequence switches to central ordering. It is a characteristic of the specific sequence (VIBE) designed by a specific vendor for the fat suppression technique. Both of them are sequential samplings of k-space and differ in the ordering within the echo train following a fat suppression pulse.

There were a number of limitations in this study. First, our study was retrospective and there was a difference in the number of patients and HCCs between both groups. Nevertheless, lesion-to-liver CNRs for each group were normally distributed and showed internally consistent patterns. Second, in the quantitative analysis, smaller lesions are affected more by the partial volume effect to measure SI using round or oval ROI. Therefore, after all HCCs were classified into two groups by short-axis diameter, CNR for small HCCs (<10 mm in the short-axis) was not measured and that for large HCCs (≥10 mm in the short-axis) was only calculated to minimize the partial volume artifacts. Detection sensitivity of FHLs was analyzed for both small and large FHLs, regardless of short-axis diameter. Finally, a few cases of HCC (4 out of 161) had Gd-EOB-DTPA uptake on HPI that resulted in iso-intensity or hyperintensity related to the background liver signal intensity. However, there was no difference in the statistical results, even though the lesions were not included in qualitative and qualitative analyses. Neither of the two readers detected 3 out of these 4 HCCs on HPI. HCCs with contrast uptake on HPI could be detected using contrast-enhanced dynamic imaging, diffusion-weighted imaging, and T2w imaging.

In conclusion, 10m-FA30 yielded diagnostic performance similar to or higher than 20m-FA10 or 20m-FA30 in patients with both compensated or decompensated chronic liver disease. This finding indicates that 10m-FA30 can potentially replace 20-min delayed HPI, regardless of patient liver function, and also reduce delay time by 10 minutes.

## Supporting Information

S1 File Attached files are data of the sensivity and lesion-to-liver CNR. (XLSX)
